# Genotypic Variation in *Trichomonas vaginalis* Detected in South African Pregnant Women

**DOI:** 10.1155/2020/1687427

**Published:** 2020-08-05

**Authors:** Rennisha Chetty, Nonkululeko Mabaso, Nathlee Abbai

**Affiliations:** School of Clinical Medicine Laboratory, College of Health Sciences, University of KwaZulu-Natal, South Africa

## Abstract

**Background:**

*Trichomonas vaginalis* is the causative agent of trichomoniasis. The genetic characterisation of *T. vaginalis* isolates reveals significant genetic diversity in this organism. Data on the prevalence of different genotypes of *T. vaginalis* in South African populations is lacking. This study investigated the diversity of *T. vaginalis* in a pregnant population in South Africa.

**Methods:**

In this study, 362 pregnant women from the King Edward VIII Hospital in Durban, South Africa, provided vaginal swabs to be tested for the presence of *T. vaginalis*. *T. vaginalis* was detected using the TaqMan assay using commercially available primers and probes specific for this protozoan (Pr04646256_s1). The *actin* gene from *T. vaginalis* was amplified with gene-specific primers. The *actin* amplicons were digested with *HindII*, *MseI*, and *RsaI*, and the banding patterns were compared across the three digests for assignment of genotypes. Phylogenetic analysis was conducted using MEGA.

**Results:**

The prevalence of *T. vaginalis* in the study population was 12.9% (47/362). Genotype G was the most frequent genotype in our study population. Genotypes H and I were detected in one sample each. According to the multiple sequence alignments and phylogenetic analysis, a level of diversity was observed across and within genotypes. Four different single-nucleotide changes in the *actin* gene were detected. Sample TV358 (H genotype) contained a single amino acid substitution from glutamine to lysine. Sample TV184 (G genotype) contained a single amino acid substitution from glutamic acid to arginine. Sample TV357 (G genotype) contained two amino acid substitutions, arginine to leucine and glycine to aspartic acid.

**Conclusion:**

Three different genotypes were observed in the pregnant population. Diversity was observed across and within genotypes. The observed diversity can be challenging for future vaccine design and development of antigen-based rapid diagnostic tests for trichomoniasis.

## 1. Introduction

*Trichomonas vaginalis* is referred to as an anaerobic, flagellated protozoan and the causative agent of trichomoniasis. This particular parasite can be transmitted via sexual intercourse from host to host [1]. Trichomoniasis is considered to be the most prevalent nonviral sexually transmitted infection (STI) worldwide affecting individuals of all ages, ethnicity, and socioeconomic groups [1–4]. The World Health Organization (WHO) estimated that in the year 2012, approximately 143 million new cases of trichomoniasis were reported in adults aged between 15 and 49 years [5]. *T. vaginalis* can infect both males and females; however, the infection rate is much higher in women [6].

Trichomoniasis has been reported to have over 276.4 million reported infections worldwide [7]. Out of the 276 million cases, 25 million cases have been reported in pregnant women [8]. Pregnant women infected with *T. vaginalis* have a higher risk of preterm delivery since *T. vaginalis* causes the premature rupture of membranes. A previous study has shown that pregnant women infected with *T. vaginalis* have a 30% risk of preterm delivery or delivering infants who have a low birth weight [9]. *T. vaginalis* infection also contributes to the increase in mother-to-child transmission of HIV [10].

Many molecular-based techniques have been used in the past in order to distinguish *T. vaginalis* strains by techniques which include microsatellite genotyping, multilocus sequence typing (MLST), polymerase chain reaction- (PCR-) hybridization, random amplification of polymorphic DNA (RAPD), and PCR-size polymorphism as well as PCR-restriction fragment length polymorphism (PCR-RFLP) [6]. Actin is one of the structural proteins present in *T. vaginalis*. This protein is well-conserved and ubiquitous in nature, thereby making it a likely option for intraspecies molecular identification [11]. To validate the use of this protein for the genetic characterisation of *T. vaginalis*, Crucitti et al. [12] conducted a study using PCR-RFLP, to identify different genotypes of *T. vaginalis* based on the *actin* gene. The study identified eight *T. vaginalis actin* genotypes among 151 isolates obtained from the Democratic Republic of Congo and Zambia [12].

The genetic characterisation of *T. vaginalis* isolates showed that there is significant genetic diversity in this organism [11]. Currently, there is a lack of data on the circulating genotypes of *T. vaginalis* in South African populations, particularly pregnant women. In this study, PCR-RFLP of the *actin* gene was performed in order to identify the different genotypes circulating in our population. Identification of the genotypes will provide a snapshot into the molecular epidemiology of *T. vaginalis* in our region which can be used as a foundation for larger epidemiological studies on this pathogen.

## 2. Methodology

### 2.1. Ethics Statement

The current study was approved by the Biomedical Research Ethics Committee (BREC) of the University of KwaZulu-Natal (BREC/00000406/2019).

### 2.2. Study Population

The study was conducted in *n* = 362 pregnant women from the antenatal clinic of the King Edward VIII Hospital in Durban, South Africa. The study was conducted from October 2018 to April 2019. Women presenting with and without symptoms of abnormal vaginal discharge were part of the study. Women presenting with symptoms were treated as part of the standard of care which was the syndromic approach for vaginal discharge syndrome. The enrolled women provided self-collected vaginal swabs for the detection of *T. vaginalis*.

### 2.3. DNA Extraction from Vaginal Swabs

Upon collection, the vaginal swab was placed in a sterile tube for molecular analysis. The samples were transported at room temperature to the School of Clinical Medicine's Research Laboratory at the Nelson R. Mandela School of Medicine, University of KwaZulu-Natal. The swabs were then resuspended in 2 ml of phosphate-buffered saline (PBS) and vortexed to remove the sample material from the swab. After vortexing, the swab was discarded and the PBS solution containing the vaginal material was subjected to DNA extraction. For the extraction, the entire 2 ml of PBS was used and the DNA was extracted using the PureLink Microbiome DNA Purification Kit (Invitrogen, supplied by ThermoFisher Scientific, United States) according to the manufacturer's instructions. The concentration and purity of the extracted DNA was assessed using the NanoDrop spectrophotometer (ThermoFisher Scientific, United States).

### 2.4. Detection of *T. vaginalis* from DNA Extracted from Vaginal Swabs

A total of *n* = 362 vaginal swab DNA samples were tested for the presence of *T. vaginalis*. *T. vaginalis* was detected using the Applied Biosystems™ TaqMan® Assays. Commercial primers and probes (Pr04646256_s1) which target the *alpha tubulin 1* gene of *T. vaginalis* were used. Amplification was performed on the QuantStudio 5 Real-Time PCR Detection System (ThermoFisher Scientific, USA). Briefly, each reaction was performed in a final volume of 5 *μ*l that comprised 0.5 *μ*l FAM-labelled probe/primer mix for individual targets, 2.5 *μ*l FastStart 4x probe master mix (ThermoFisher Scientific, Pr04646256_s1), 1.5 *μ*L template DNA, and nuclease-free water. We also included nontemplate control reactions. Amplification was performed under the following conditions: 1 cycle at 95°C for 30 seconds followed by 45 cycles of denaturation at 95°C for 3 seconds and annealing at 60°C for 30 seconds. The detection of fluorescent products was performed at the end of the annealing period. The raw fluorescent data that included the *C*_T_ mean values were automatically generated by the QuantStudio 5 Real-Time PCR System software.

### 2.5. Detection of the *Actin* Genes from *T. vaginalis*

A conventional nested PCR assay was used for the amplification of the *actin* genes (outer and inner regions) using oligonucleotide primers, published by Espinosa et al. [13] and Khalili *et al*. [6]. The primers used for amplification of inner and outer *actin* genes in one woman are shown in Table 1.

### 2.6. Amplification of the Outer *Actin* Gene

The amplification reactions were performed in PCR with a total volume of 25 *μ*l. The reaction contained 12.5 *μ*l DreamTaq master mix (ThermoFisher Scientific, Massachusetts, United States), 9.5 *μ*l distilled water, and 0.5 *μl* of each primer (reverse and forward), and 2 *μ*l of no-template PCR product was used. The negative control contained 23 *μ*l of PCR mixture and 2 *μ*l of distilled water. Thereafter, the PCR tubes were placed into the thermal cycler and the following conditions were performed, for gene amplification, initial denaturation at 94°C for 5 minutes, thereafter 30 cycles: denaturation at 94°C for 1 minute, annealing 54°C for 1 minute, elongation 72°C for 1 minute, and final elongation at 72°C for 5 minutes.

### 2.7. Amplification of the Inner *Actin* Gene by Nested PCR

The nested amplification reactions were performed in PCR with a total volume of 25 *μ*l. The reaction contained 12.5 *μ*l DreamTaq master mix, 9.5 *μ*l distilled water, 0.5 *μ*l of each primer (reverse and forward) and 2 *μ*l of outer PCR product. The negative control contained 23 *μ*l of PCR mixture and 2 *μ*l of distilled water. Thereafter, the PCR tubes were placed into the thermal cycler and the following conditions were performed: for gene amplification, initial denaturation at 94°C for 5 minutes, thereafter 30 cycles: denaturation at 94°C for 1 minute, annealing 45°C for 1 minute, elongation 72°C for 1 minute, and final elongation at 72°C for 5 minutes.

### 2.8. Sequence Confirmation of the *Actin* Gene

A subset of PCR positive amplicons was sequenced to confirm the presence of the gene prior to the genotyping analysis. Sanger DNA sequencing was performed on the inner *actin* PCR amplicons. Each amplicon was sequenced in both directions to cover the full-length *actin* gene. The sequencing was conducted using the BrilliantDye™ Terminator v3.1 Cycle Sequencing on an ABI3500XL genetic analyser. The sequencing was performed at Inqaba Biotechnical Industries (Hatfield, Pretoria, South Africa). The ABI sequencing files were edited on CHROMAS (Technelysium, Queensland, Australia). The forward and reverse sequences were aligned using the DNAMAN software (Lynnon Biosoft, California, United States). The identity of the edited sequences was confirmed using the National Center for Biotechnology Information (NCBI) Basic Local Alignment Search Tool (BLAST).

### 2.9. Restriction Fragment Length Polymorphisms (RFLP)

The genotyping of the actin genes was performed using the RFLP technique. Restriction enzymes, *HindII*, *Mse*I, and *Rsa*I were used to generate banding profiles. The inner actin amplicons were digested with the individual enzymes. The digestion mix was made up to a final volume of 20 *μ*l. Each reaction consisted of 0.5 *μ*l enzyme, 2 *μ*l enzyme buffer, 0.2 *μ*l bovine serum albumin (BSA), 7.3 *μ*l distilled water, and 10 *μ*l of the PCR amplicon. The digestion reactions were incubated for 4 hours under the following temperature conditions: 37°C for both *MseI* and *RsaI* enzymes and 64°C for the *HindII* restriction enzyme [6]. Following incubation, the digests were run on a 2% agarose gel which were stained with 4 *μ*l of SYBR Safe dye (ThermoFisher Scientific, Massachusetts, United States). Each well was then loaded with 5 *μ*l of loading dye and 20 *μ*l of the digestion mix. The gels were electrophoresed at 80 V for 2 hours. The enzyme's banding patterns and assignment of genotypes based on a composite of the patterns were determined according to Khalili et al. [6].

### 2.10. Phylogenetic Analysis of *Actin* Genotypes

Selected samples harbouring different genotypes for the *actin* gene after digestion with the 3 enzymes were selected for further phylogenetic analysis. The amplicons were sequenced in both directions using the Sanger approach. The sequencing was performed on an ABI3500XL genetic analyser at Inqaba Biotechnical Industries (Hatfield, Pretoria, South Africa). The ABI sequencing files were edited on CHROMAS (Technelysium, Queensland, Australia). The forward and reverse sequences were aligned using the DNAMAN software (Lynnon Biosoft, California, United States). The DNA sequences were translated to protein using the freely available software ExPASy (https://web.expasy.org/translate/). The translated sequences together with published actin protein sequences were aligned using ClustalW (https://www.genome.jp/tools-bin/clustalw). The alignments were performed in order to identify amino acid substitutions. A phylogenetic tree was then constructed from the sequence data using the Molecular Evolutionary Genetics Analysis (MEGA) version 10 software (Arizona, United States).

## 3. Results

### 3.1. Detection of *T. vaginalis* from DNA Extracted from Vaginal Swabs

The presence of the *alpha tubulin 1* gene was detected in 47/362 swab DNA samples by the real-time PCR TaqMan assay. The prevalence of *T. vaginalis* in this population was 12.98. The quantification cycle (*C*_q_) for the samples that produced positive amplification ranged from 25 to 35 cycles. All negative no-template controls did not produce any amplification.

### 3.2. Amplification of the Outer and Inner *Actin* Genes of *T. vaginalis*

**Figure 1:**
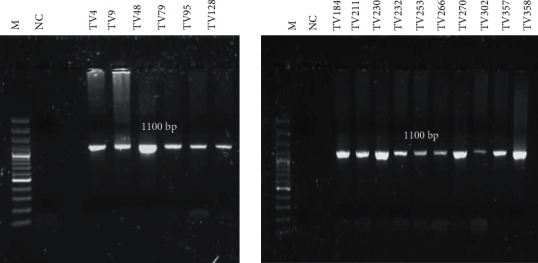
Agarose gel showing positive amplicons generated for the inner *actin g*ene. The expected fragment size of 1100 bp was observed. M: 100 bp DNA molecular ladder (ThermoFisher Scientific), NC: negative control (no template DNA added), and amplified clinical samples. A product size of 1100 bp indicative of the inner *actin* gene was present in all 16 samples tested.

Amplification of the outer *actin* gene by conventional PCR yielded 16 positives out of the 47 samples tested. The majority of the samples (23/31) that did not test positive in the initial PCR showed either low DNA concentration or purity ratios. For the low-concentration samples, the reactions were repeated with an increased amount of DNA; however, the adjustment still did not result in successful amplification of the outer *actin* gene. The inner *actin* gene was amplified from all 16 samples. The expected fragment size of 1100 bp was observed by agarose gel electrophoresis (Figure 1). The DNA sequencing hits of the *actin* gene showed identity (99%) to *T. vaginalis isolate 19 actin gene* (MF350343.1) and *T. vaginalis strain ATCC 30240 actin gene* (99%) (EU076579.1).

### 3.3. Genotyping Analysis

#### 3.3.1. HindII Profile

**Figure 2:**
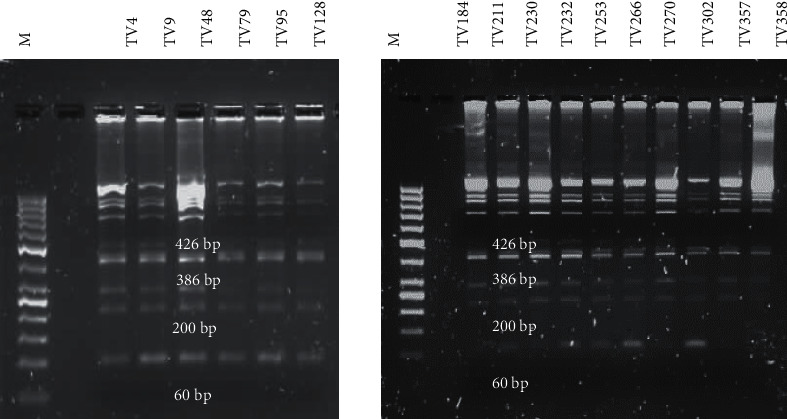
*HindII* RFLP pattern of the digested *actin* gene amplicon resolved on a 1.5% agarose gel. M: O'GeneRuler 50 bp DNA Ladder (ThermoFisher Scientific) and banding profiles of the clinical samples. Size fragments of 60 bp, 200 bp, 386 bp, and 426 bp were observed for the respective samples.

All samples produced a banding pattern for *HindII*. A similar banding profile was observed across all samples (Figure 2). Numerous bands were observed for this digest which had not been previously published [6]. Since all the samples produced the same banding profile, an in silico analysis was performed on nine samples for which the full sequences of the *actin* gene were available using the freely available Restriction Mapper tool (http://www.restrictionmapper.org/cgi-bin/sitefind3.pl). The in silico data for one sample (TV266) is shown in Table 2. According to Restriction Mapper, only four fragments should have been yielded at positions 60 bp, 200 bp, 386 bp, and 426 bp (Table 2). These four fragments were shown to be present on the gel (Figure 2). The additional bands observed could have been due to star activity by the enzyme (i.e., cutting at nonspecific recognition sites due to prolonged incubation period or too high enzyme concentration). Based on the identical banding pattern across all samples, the samples were assigned the same pattern code, i.e., pattern 2 (Table 3). The in silico analysis that was performed on the nine samples did not alter the assignment of the genotypes; it confirmed the genotype assignments based on the *HindII* digests.

#### 3.3.2. RsaI Profile

**Figure 3:**
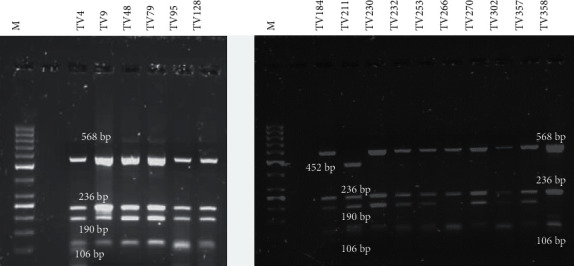
*Rsa*I RFLP pattern of the digested *actin* gene amplicon resolved on a 1.5% agarose gel. O'GeneRuler 50 bp DNA Ladder (ThermoFisher Scientific) and banding profiles of the clinical samples. Fragment sizes of 106 bp, 190 bp, 236 bp, 452 bp, and 568 bp were observed.

All samples produced banding patterns for *RsaI*. Three different banding patterns were observed for *RsaI* (Figure 3). Of the 16 samples digested, 13/16 (81.25%) of the samples produced four fragment sizes at positions 106 bp, 190 bp, 236 bp, and 568 bp. This group of samples were assigned the code pattern 1. Two of the 16 samples (12.50%) produced three fragment sizes at positions 106 bp, ^2^36 bp, and 568 bp, and the one sample (6.25%) produced four fragments at positions 106 bp, 190 bp, 236 bp, and 452 bp. The groups were assigned the codes patterns 2 and 3, respectively (Table 3). The size of the digestion fragments obtained corresponded to previous studies that reported on genotyping of the *actin* gene by *RsaI* digestion.

#### 3.3.3. MseI Profile

**Figure 4:**
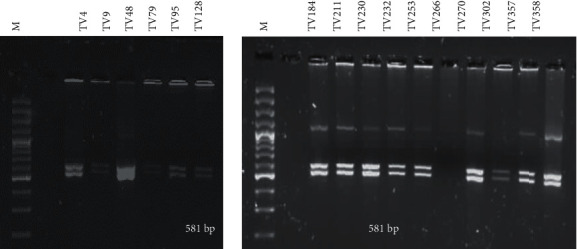
*Mse*I RFLP pattern of the digested *actin* gene amplicon resolved on a 1.5% agarose gel. M: 100 bp DNA ladder (ThermoFisher Scientific) and banding profiles of the clinical samples. Fragment sizes of 519 bp and 581 bp were observed.

For the *MseI* reactions, one sample did not produce a banding pattern despite numerous adjustments such as increased incubation time and increased amounts of template DNA. The sample produced a very faint uncut band. For the remaining 15/16 (93.75%) of the samples, an identical banding profile was observed for all the samples. Bands at positions 519 bp and 581 bp were observed (Figure 4). All of the samples producing a digestion pattern were assigned the code pattern 1 (Table 3).

### 3.4. Frequency of Genotypes

According to the combined pattern codes for all three enzymes, the G genotype was the most frequent genotype in our study population. A total of 13/16 (81.25%) of the samples harboured this genotype. One sample (TV358) harboured genotype H (6.25%). Similarly, one sample (TV211) harboured genotype I (6.25%). There was one sample (TV266) that could not be assigned a genotype since it lacked a banding profile for *MseI*; a pattern code could therefore not be determined. This sample was therefore excluded from further sequence analysis (Table 3).

### 3.5. Phylogenetic Analysis of the *Actin* Gene Open Reading Frame

**Figure 5:**
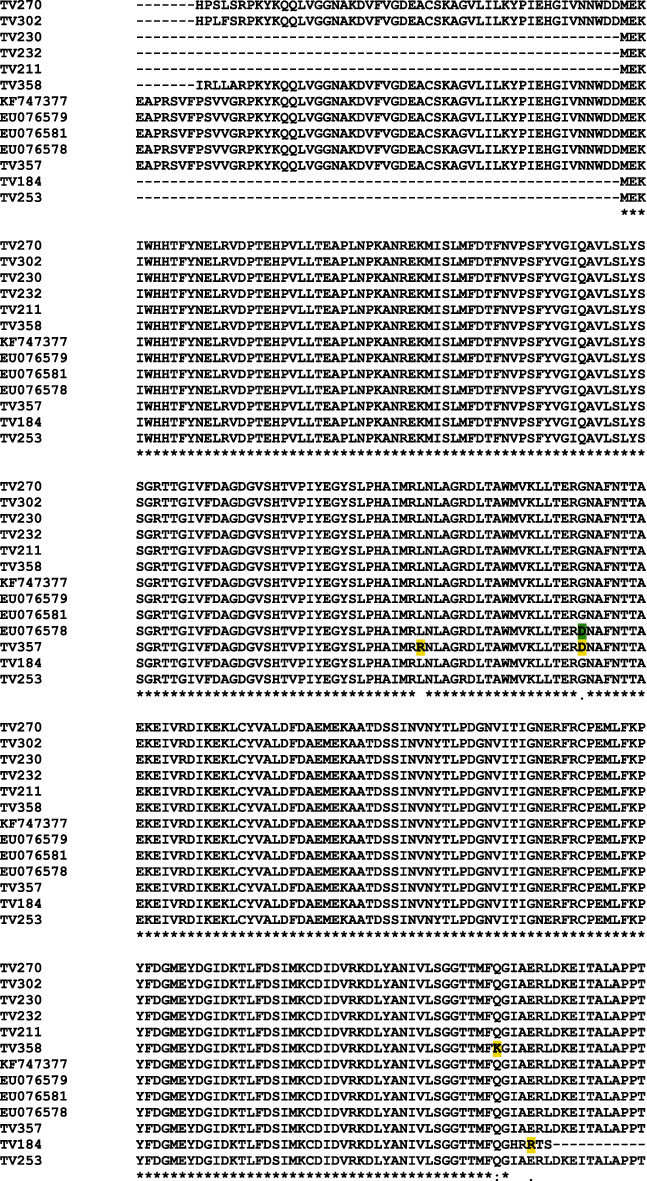
Multiple sequence alignment of the actin ORF from clinical samples and published genotypes using ClustalW (GenomeNet). A total of 4 different single-nucleotide changes in the open reading frame (ORF) of the *actin* gene were detected for the clinical samples (changes highlighted in yellow). A single change in one of the published sequences was also noted (highlighted in green).

Translated nucleotide sequences obtained for the *actin* gene from selected clinical samples were edited and aligned with published sequences for genotypes G and H. There were no published sequences for genotype I to include in this analysis. According to the multiple sequence alignment, a total of four different single-nucleotide changes in the open reading frame (ORF) of the *actin* gene was detected (Figure 5). Sample TV358 (genotype H) contained a single amino acid substitution from glutamine (Q) to lysine (K). Sample TV184 (genotype G) contained a single amino acid subsititution from glutamic acid (E) to arginine (R). Sample TV357 (genotype G) contained two amino acid substitutions. The first change was the replacement of leucine (L) with arginine (R), and the second change was the replacement of GLYCINE (G) with aspartic acid (D). The change from G→D was also observed for a published *T. vaginalis ATCC* genotype G strain (EU076578) (Figure 5).

**Figure 6:**
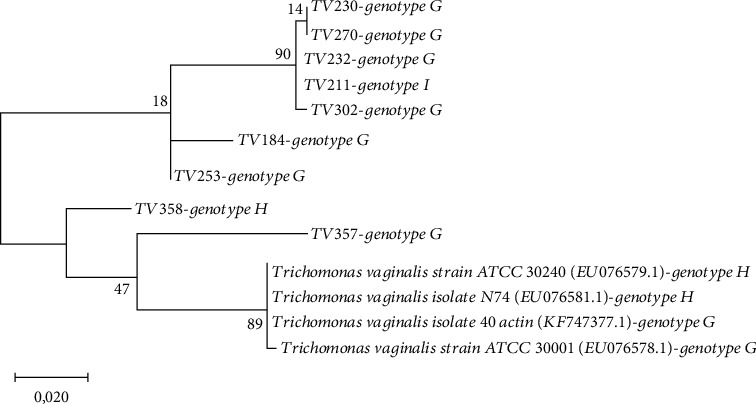
Phylogenetic tree analysis of *Trichomonas vaginalis* actin genotypes according to the maximum-likelihood (ML) tree was conducted based on the multiple sequence alignment of actin gene by MEGA V10. Distance represents the number of base substitutions per site.

The phylogenetic analysis of the *actin* ORF from the clinical samples and published genotypes showed the presence of distinct clusters. Overall, the majority of the samples analysed in this study were not closely related to any of the published sequences with the exception of samples TV357 and TV358 (Figure 6). The tree contained one cluster with only the clinical samples from genotype G (4 samples) and the one sample from genotype I. This indicates that there was no significant diversity in the *actin* ORF of genotype I and certain genotype G samples. However, within the group of genotype G samples, a level of diversity was noted. Samples TV184, TV253, and TV357 did not cluster with the other genotype G samples. TV184 contained a single amino acid substitution (E→R) which was not present in the other genotype G samples. Sample TV253 did not contain any substitutions; however, it did not cluster with the other samples of genotype G without substitutions. Sample TV357 was shown to cluster closely to the published G and H genotypes. This sample also carried two amino acid substitutions in the *actin* gene ORF (L→R) and (G→D). One of these substitutions (G→D) was also observed for one of the published genotype G strains (EU076578). Sample TV358 (genotype H) clustered on its own. This sample also contained a substitution (Q→K) in the *actin* gene ORF (Figure 5).

### 3.6. Distribution of Genotypes in Relation to Clinical Symptoms

Overall, the median age of the women who tested positive for *T. vaginalis* in this study was 28.0 (24.0-32.5). The majority of the study women had completed high school (68.1%), were unmarried (91.5%), had a regular sex partner (80.9%), had between 2 and 4 lifetime sex partners (66.0%), and were in the last trimester of pregnancy (59.6%) (data not shown).

For the one woman who carried genotype I, it was shown that this woman reported multiple symptoms associated with STIs such as abnormal vaginal discharge, foul-smelling vaginal odour, genital itching, and genital warts (Table 4). The one woman harboured genotype H was asymptomatic (Table 4). For the women who harboured genotype G, it was shown that a large proportion of these women (53.8%) were asymptomatic, followed by 23.0% who reported symptoms of abnormal vaginal discharge, 15.3% who reported genital itching, and 7.6% who reported a combination of symptoms (abnormal vaginal discharge and genital itching (Table 4).

## 4. Discussion

Trichomoniasis is considered to be the most prevalent nonviral sexually transmitted infection (STI) worldwide affecting individuals of all ages, ethnicity, and socioeconomic groups [2, 3]. The global prevalence rates for *T. vaginalis* varies between 0.9% and 80% [14–16].

In this study, the prevalence of *T. vaginalis* in antenatal women was approximately 13.0%. The risk factors associated with infection in this cohort which have been described elsewhere included previous history of STIs and current abnormal vaginal discharge [17]. A similar prevalence rate of 10% for *T. vaginalis* was observed for antenatal women from Durban [18]. The prevalence estimates reported in South African women correlate with the rates reported for other African countries. A study conducted by Abou-Kamar *et al.* [16] in Egypt on pregnant and nonpregnant women showed a prevalence rate of 13.5% for *T. vaginalis*. An earlier study conducted by the WHO in the African region in women aged 15 to 49 years of age reported an estimated prevalence rate of 11.5% for *T. vaginalis* infection [19].

Early techniques which were applied for the typing of *T. vaginalis* isolates included; antigenic characterisation [20], monoclonal antibody binding [21], karyotype polymorphism by pulsed-field gel electrophoresis [22] [23], random amplified polymorphic DNA (RAPD) [24], and restriction fragment length polymorphisms (RFLP) [25]. Despite the numerous list of typing techniques, each of the abovementioned had significant limitations [11].

Currently, the PCR-RFLP technique based on the *actin* gene amplification provides a sensitive and reliable method for typing of *T. vaginalis* isolates [26]. In this study, three different genotypes of *T. vaginalis* were identified by PCR-RFLP of the *actin* gene. The most frequent genotype in our study population was the genotype G. Other *T. vaginalis* genetic studies conducted on the African continent revealed the presence of genotypes G and E as highly prevalent [12] [27],. A study conducted in the Democratic Republic of Congo (DRC) and Zambia on female sex workers revealed the presence of eight different genotypes based on the *actin* gene. In the DRC, the most prevalent genotype was E, whereas in Zambia the most common genotype was G [12]. Our study findings are consistent with the findings from Zambia. A study conducted on pregnant women from Kenya reported a high frequency of genotype E [27] which is similar to the results obtained for the DRC [12]. From the abovementioned studies, it is evident that common genotypes of *T. vaginalis* are circulating in various population groups across the African continent. In a study conducted in Ndola, Zambia, involving adolescent girls and pregnant women as well as sex workers, Crucitti et al. [28] identified nine different genotypes, with genotype G being the most frequent across all three study groups. This is similar to the findings of the pregnant women tested in the current study.

In studies conducted out of Africa, Momeni et al. [29] identified five different genotypes with genotype G being the most prevalent in a population of Iranian men and women. However, three recent studies conducted in Iran [6] [26], [30], observed a high prevalence of *T. vaginalis* genotypes which differed from the genotypes observed in Africa and those reported by Momeni *et al*. [29] for Iran. Matini *et al*. [30] investigated the prevalence of *T. vaginalis* genotypes in symptomatic and asymptomatic women, attending gynaecology clinics in western Iran. According to the *actin* gene digestion profiles, genotype A was shown to be the most prevalent in their study population [30]. A study conducted on women seeking care in a general clinic in southwestern Iran reported on genotype H as being the most frequent [6]. Similar findings were reported by Oliaee *et al*. 2017 [26] in a population of incarcerated women from southern Iran where genotype H was shown to be the most frequent [26].

In this study, within the samples that belonged to genotype G, a level of diversity was noted as evidenced by the multiple sequence alignments and phylogenetic analyses. Three samples TV184, TV253, and TV357 did not cluster with the other genotype G samples. The possibility of obtaining different genotypes involving *T. vaginalis* within a study population could be due to large sample numbers as well as obtaining samples from regions which have a much higher prevalence rate [16]. In this study, the prevalence of *T. vaginalis* was close to 13% and this relatively high prevalence could have contributed to the observed diversity. In addition, for the women who carried genotype G, it was shown that a large proportion of these women (53.8%) were asymptomatic, followed by 23.0% who reported symptoms of abnormal vaginal discharge, 15.3% who reported genital itching, and 7.6% who reported a combination of symptoms (abnormal vaginal discharge and genital itching). A study conducted by Khalili et al. [6] also showed that in the group of women who carried genotype G, there was a combination of women who were asymptomatic as well as symptomatic. It is possible that the genetic differences in the pathogen may contribute to the clinical manifestations associated with infection. However, this will need to be confirmed by further investigations.

Within the genotype G study samples, two of the three samples carried amino acid substitutions in the *actin* gene ORF. One particular sample TV357 showed a replacement of leucine (L) with arginine (R) and replacement of glycine (G) with aspartic acid (D). Similar findings were reported by Spotin *et al*. [31] for a *T. vaginalis* isolate from Iran. The multiple alignment of the Iranian isolate which was assigned genotype G showed amino acid substitutions where aspartic acid (D) replaced glycine (G) and arginine (R) replaced leucine (L) [31]. This indicates that there may be a small level of similarity between *T. vaginalis* genotype G samples from different regions. Identifying isolates from different geographical regions which are closely related is beneficial to future vaccine development studies for this pathogen.

## 5. Limitations

In this study, the *actin* gene was not shown to be present in all the swab samples which tested *T. vaginalis* positive by the TaqMan assay. Other *T. vaginalis* genotyping studies which have been based on the *actin* gene were conducted on pure isolates rather than the primary vaginal swab sample. The use of the primary sample to infer genotypes may not be the most appropriate method as evidenced by the lack of amplification of certain samples. An alternate method which may be more useful for investigating the genetic variation in *T. vaginalis* from noncultured vaginal swabs would be the next generation-multilocus sequence typing (NG-MLST) approach described by Squire et al. [32]. However, due to budget constraints, the NG-MLST method was not used in the present study.

## 6. Conclusion

The present study provides evidence on the genetic diversity of *T. vaginalis* from a South African pregnant population. Three different genotypes were observed in the studied population. Within the genotype G samples, diversity in the *actin* gene ORF was observed. The observed diversity within specific populations can be challenging for future vaccine design and development of antigen-based rapid diagnostic tests for trichomoniasis. However, one of our South African isolates was closely related to an Iranian isolate. This holds some promise that there may be conserved *T. vaginalis* isolates from different geographical regions. This could lend some hope for future vaccine design and diagnostic studies focused on identifying antigenic determinants which are broadly representative of the entire *T. vaginalis* population [11].

Future work emanating from this study would be to investigate the association of the genotypes with patterns of drug susceptibility in this pathogen. In addition, it would be useful to compare the distribution of the genotypes in pregnant and nonpregnant populations as well as look at the association of the genotypes with the prevalence of the different *T. vaginalis* viruses (TVVs).

## Figures and Tables

**Table 1:** Description of primers used for the amplification of the *actin* genes in the studied population.

Primer name	Primer sequence
Outer actin forward (Tv8S)	5′-TCTGGAATGGCTGAAGAAGACG-3′
Outer actin reverse (Tv9R)	5 ′-CAGGGTACATCGTATTGGTC-3′
Inner actin forward (Tv10S)	5′-CAGACACTCGTTATCG-3′
Inner actin reverse (Tv11R)	5′-CGGTGAACGATGGATG-3′

**Table 2:** Virtual digest of sample TV266 with the Restriction Mapper tool (http://www.restrictionmapper.org/cgi-bin/sitefind3.pl).

Length	5′ enzyme	5′ base	3′ enzyme	3′ base	Sequence
426	*HindII*	261	*HindII*	686	GACCCAACAG AGCACCCAGT TCTTCTTACA GAAGCCCCAC TCAACCCAAAGGCTAACCGT GAGAAAATGA TCTCCCTCAT GTTCGACACATTCAATGTCCCATCCTTCTA TGTCGGCATC CAGGCTGTTCTTTCCCTCTA CTCCTCTGGCCGTACAACAGGTATCGTTTT CGATGCTGGT GATGGTGTTT CCCACACAGTTCCAATTTAC GAAGGCTACT CCCTTCCACA CGCCATCATG AGACTTAACCTCGCTGGCCG TGATCTCACA GCCTGGATGG TCAAGCTTCT CACAGAGCGTGGCAATGCTT TCAACACAAC AGCCGAAAAG GAAATCGTTC GTGACATCAAGGAGAAGCTT TGCTATGTCG CCCTCGACTT CGATGCTGAA ATGGAGAAGGCCGCTACAGA CTCCTCCATC AACGTC

386	*HindII*	687	None	1072	AACTACACAC TTCCAGATGG CAACGTCATC ACAATCGGCA ATGAGCGCTTCCGCTGCCCA GAAATGCTCT TCAAGCCATA CTTCGATGGT ATGGAATACGATGGTATCGA CAAGACACTC TTCGACTCCA TCATGAAGTG CGATATCGATGTTCGTAAGG ATCTCTACGC TAACATCGTT CTTTCTGGTG GCACAACAATGTTCCAAGGG CATCGCCGAA CGTCTTGACA AGGAAATCAC AGCTCTTGCTCCACCAACAA TGAAGGTCAA GATCGTCGCC CCAGAAGAGC GTAAGTACGCCGTTTGGGTC GGTGGCTCCA TCCTTGCTTC CCTCGCTACA TCCCACAGATGGGTATCACA AGGAGGAATA CGACGAGGCT GGTCTC

200	None	1	*HindII*	200	ACGATACGCT CTGGTATGTG CAAGACGGCT TCTCTGGCGA TGAAGCCCCACGCTCTGTTT TTCCCATCCG TTGTTGGCCG ATCCAAAAGT ACAAACAACAATTAGTTGGT GGCAACGCCA AGGATGTCTT CGTCGGTGAT GAAGCTTGCTCCAAGGCTGG TGTCCTCATC CTCAAGTACC CAATTGAACA CGGTATCGTC

60	*HindII*	201	*HindII*	260	AACAACTGGG ATGATATGGA AAAGATCTGG CACCACACAT TCTACAACGAACTTCGTGTT

**Table 3:** Fragment sizes obtained after digestion of the *actin* gene with the restriction enzymes *HindII*, *MseI*, and *RsaI* as well as the assignment of the *T. vaginalis* genotypes based on combining the patterns across the three enzyme profiles.

Sample name	*HindII* fragment sizes	*HindII* pattern	*MseI* fragment sizes	*MseI* pattern	*RsaI* fragment sizes	*RsaI* pattern	Genotype
TV4	60, 200 386, 426	2	519, 581	1	106, 190, 236, 568	1	G
TV9	60, 200 386, 426	2	519, 581	1	106, 190, 236, 568	1	G
TV48	60, 200 386, 426	2	519, 581	1	106, 190, 236, 568	1	G
TV79	60, 200 386, 426	2	519, 581	1	106, 190, 236, 568	1	G
TV95	60, 200 386, 426	2	519, 581	1	106, 190, 236, 568	1	G
TV128	60, 200 386, 426	2	519, 581	1	106, 190, 236, 568	1	G
TV184	60, 200 386, 426	2	519, 581	1	106, 190, 236, 568	1	G
TV211	60, 80, 120, 200 330, 426	2	519, 581	1	106, 190, 236, 452	3	I
TV230	60, 200, 426, 449	2	519, 581	1	106, 190, 236, 568	1	G
TV232	60, 200 399, 426	2	519, 581	1	106, 190, 236, 568	1	G
TV253	60, 208 397, 426	2	519, 581	1	106, 190, 236, 568	1	G
TV266^#^	60, 200 386, 426	2	-^∗^		106, 236, 568	2	Could not be assigned
TV270	60, 202 387, 426	2	519, 581	1	106, 190, 236, 568	1	G
TV302	60, 200 390, 426	2	519, 581	1	106, 190, 236, 568	1	G
TV357	60, 201 387, 426	2	519, 581	1	106, 190, 236, 568	1	G
TV358	60, 199 388, 426	2	519, 581	1	106, 236, 568	2	H

∗(-) symbol indicates that no banding pattern was observed. ^#^Genotype could not be assigned due to no banding pattern for *MseI*.

**Table 4:** Genotypes linked to clinical symptoms of infection.

Genotype	Number of isolates (%)	Clinical symptoms reported
I	1/1 (100%)	Abnormal vaginal discharged, foul-smelling vaginal odour, genital itching, and genital warts
H	1/1 (100%)	No symptoms
G	7/13 (53.85%)	No symptoms
G	3/13 (23.08%)	Abnormal vaginal discharged
G	2/13 (15.38%)	Genital itching
G	1/13 (7.69%)	Abnormal vaginal discharged and genital itching

## Data Availability

The data used to support the findings of this study are available from the corresponding author upon request.
